# Social norms that sustain transactional sex and associations with sexual health outcomes: A mixed-methods study in the Comarca Ngäbe-Buglé, a rural-Indigenous region of Panama

**DOI:** 10.1371/journal.pone.0304805

**Published:** 2024-05-31

**Authors:** Amanda Gabster, Philippe Mayaud, Mónica Jhangimal, Juan Miguel Pascale, Suzanna C. Francis, Ben Cislaghi

**Affiliations:** 1 Instituto Conmemorativo Gorgas de Estudios de la Salud, Panamá City, Panamá, Panamá; 2 London School of Hygiene and Tropical Medicine, London, United Kingdom; 3 Sistema Nacional de Investigación, SENACYT, Panamá City, Panamá, Panamá; 4 Center of Population Sciences for Health Equity, Florida State University, Tallahassee, FL, United States of America; 5 Facultad de Medicina, Universidad de Panamá, Panamá City, Panamá, Panamá; Universidad Nacional Autónoma de México Facultad de Medicina: Universidad Nacional Autonoma de Mexico Facultad de Medicina, MEXICO

## Abstract

The Comarca Ngäbe-Buglé (CNB), home to >200,000 Indigenous people, is one of the poorest regions in Panama. We describe transactional sex (TS) behaviours, normative beliefs and factors associated with TS among Indigenous adolescents(14-19years) in the CNB. We conducted a mixed-methods study in the CNB between January and November 2018, which included a qualitative study with participant observation and semi-structured interviews that focused on descriptive norms related to TS; and a cross-sectional study among public-school-going adolescents using self-administered questionnaire to report sexual behaviour and injunctive norms related to TS. Participants in the epidemiological study were also asked to submit samples for HIV, syphilis, chlamydia, and gonorrhoea testing. Qualitative thematic analysis was used to organise and analyse field notes and semi-structured interviews. Quantitative analysis included four models: TS experience and acceptance of a TS offer and the associations of these outcome variables with demographic and behavioural variables and HIV/STI infections. In the qualitative study among 20 adolescents, we found that people offering TS were reported to be from within and outside of the community, and included older men and women, and disturbingly, teachers. Participants reported feeling individual and collective agency in the decision to engage in TS and described little social sanctions for participation. In the quantitative study among 700 adolescents(309 girls[45.1%],379 boys[54.9%]), we found that girls(18.8%;58/309) and boys(15.5%;58/379) reported similar levels of having been offered TS, and of acceptance among those offered(girls 81.4% [35/43]; boys 77.8% [35/45]). TS was found to be associated with the reported forced sex and HIV/syphilis seropositivity. Due to widespread acceptance and feelings of agency, interventions would not be effective if they focused on eliminating the transactional component of sexual encounters. Instead, interventions should focus on individual and household economic stability, increasing violence reporting, bringing perpetrators to justice, and adopting condom use during all sexual encounters.

## Introduction

Transactional sex (TS) has been broadly defined as “the implicit exchange of money or material goods for sex” [1–4]. Motivations for engaging in TS range from 1) fulfilling basic needs, 2) seeking improved social status, and 3) material expression of love [4]. In the 1990s, social scientists and medical literature began to differentiate commercial sex work from transactional sex due to the potentially stigmatising labels of sex work [5]. A recently proposed definition of TS includes ´noncommercial, non-marital sexual relationships motivated by the implicit assumption that sex will be exchanged for material support or other benefits´ [4]. Although TS has often been conflated with commercial sex work in research, distinctions have been made: someone who participates in sex work self-identifies as a sex worker, explicitly exchanges money or goods and with little or no emotional intimacy with the person offering, while someone who participates in TS does not self-identify as a sex-worker, the exchange of money or goods is implicit and there is some emotional intimacy [5].

Research on TS among adolescent populations has focused on young women’s vulnerability in sub-Saharan Africa. TS has been associated with poor sexual health outcomes and risks in these studies, including HIV and sexually transmitted infections (STIs) [6–12]. Studies in Latin America have highlighted associations between TS and HIV among men who have sex with men (MSM) and transwomen populations [13,14]. In Latin America, two studies among Indigenous women found syphilis positivity to be associated with TS [15,16]. However, there is no prior research of TS practices and norms among Indigenous adolescents in Latin America.

Few studies have investigated social norms that influence TS activity among adolescents. Social Norms Theory explains how peers influence adolescents’ behaviour. During adolescence, behavioural guidance shifts from caregivers to peers, especially same-sex peers [17]. Due to this, the reference group for social norms research among adolescent populations is often the peer group. Social norms can be either descriptive or injunctive: descriptive norms are beliefs people hold about what they think others do in a situation; injunctive norms are what people think others in their reference group approve or disapprove of [18]. Social sanctions, the anticipation of reward or punishment from the reference group, hold norms in place [19]. Culture, a normative system of shared values, holds normative behaviours in place over time through socialization of behaviours [20,21]. Despite the hundreds of distinct cultural groups within Latin America and the Caribbean, there is little prior research into how social norms affect TS among adolescents outside of sub-Saharan Africa, especially among Indigenous adolescents in Latin America. By understanding who engages in TS, who offers TS, the normative beliefs surrounding the practice, and the sexual health outcomes associated with TS, relevant and more effective interventions could be designed.

Across Latin America, Indigenous populations are at higher risk of acquiring HIV than non-Indigenous populations [22]. In Panama, Indigenous people make up over 12% of the total population; there are six ´Comarcas´ across the country, which are semi-politically autonomous Indigenous administrative regions. The Comarca Ngäbe-Buglé (CNB), home to 5.1% (>200,000 individuals) of the Panamanian population, is the largest Comarca in geographical size and population [23]. In 2017, the CNB had a multidimensional poverty index of 93.4%: the country’s highest poverty level (national average: 19.1%) [24]. Prior to this research, there were limited data on HIV and STIs among adolescents of the CNB. However, the 2018 epidemiological data showed that new diagnoses of HIV among adolescents (15–19 years) of the CNB accounted for 20.5% of adolescent diagnoses nationwide (13.2% of adolescent female and 25.7% of adolescent male diagnoses) [25].

This analysis of TS among adolescents of CNB was part of a broader study to determine the prevalence and risk factors of HIV and other STIs among CNB adolescents. Transactional sex has not previously been studied among Indigenous adolescents in Panama. In this study, we use qualitative and quantitative methods to describe some factors associated with TS practices, normative beliefs surrounding TS activity, and the association of TS with adverse sexual health outcomes among adolescent boys and girls of the Comarca Ngäbe-Buglé. Understanding these factors is imperative for designing sexual health interventions for this population.

## Methods

### Study design

We conducted a mixed-methods study to describe the normative beliefs around TS and the association with sexual health outcomes [26]. We integrated qualitative and quantitative research in two instances: 1) at the design level and 2) at the interpretation level. At the design level, as little was known about the sexual behaviours and STIs of adolescents of the CNB, we used an exploratory sequential framework in which qualitative methods were first employed; quantitative methods were then used to measure the generalizability and prevalence of qualitative findings and HIV/STI outcomes [27]. At the interpretation level, we used the weaving approach in the discussion to describe qualitative and quantitative findings by major points of discussion [27].

#### Qualitative methods

**Fig 1:**
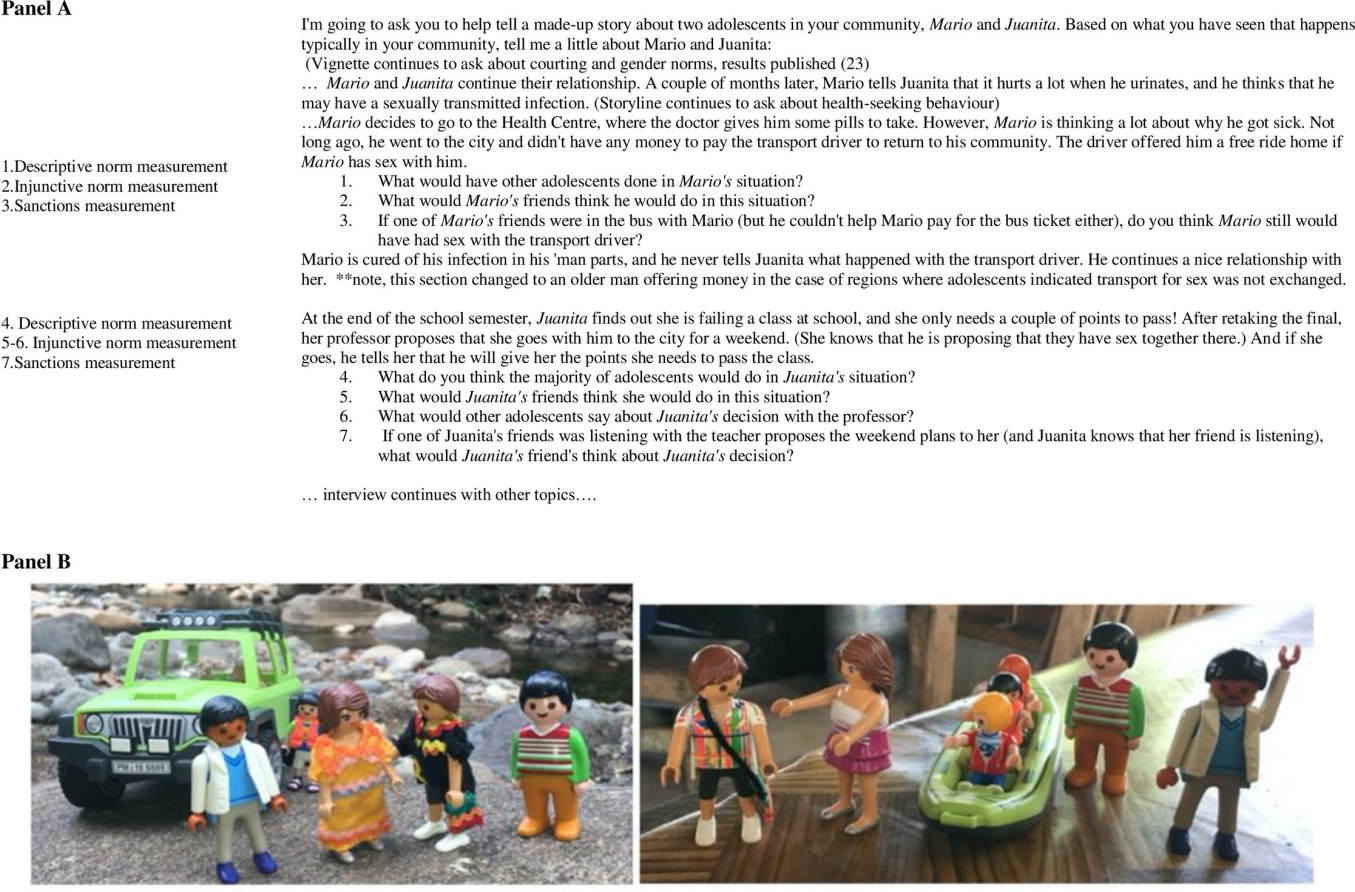
Semi-structured vignette example (Panel A), and dolls used to act out vignette (Panel B).

The qualitative study (including participant observation and semi-structured interviews) was conducted in two communities which were similar in size (approximately 2,500 inhabitants) and population density, one on either side of the Central Mountain Range in the CNB [28]. In each community, AG undertook participant observation over a one-month period. the community and researcher would get to know each other and to purposively select participants. This participant observation included living in the community with families as well as sharing life and domestic chores. During that same month, semi-structured interviews were undertaken after an initial 10-day participant observation period. Both communities were part of the quantitative study. Therefore, some participants could have partaken in both studies. The interviews used vignettes to elicit descriptive norms and social sanctions. Twenty adolescents (5 boys and 5 girls from each community) were purposively selected during the participant observation based on potential quality interviews (volunteering to speak to AG about research topics, ease of speaking about topics related to sexuality, and based on gender of potential participant). There were twenty interviews were performed as thematic saturation for all qualitative analyses was reached at this point (two other qualitative studies with separate aims have been published elsewhere [29,30]). Briefly, semi-structured interviews, undertaken by author AG in Spanish, were 30–60 minutes long and were undertaken in a private location of the interviewee’s choice. During the interview, consent/assenting participants were asked to use dolls to act out a vignette that described the descriptive norms of adolescents’ peers regarding sexual decision-making. Questions related to TS and social norms are found in **Fig 1, Panel A;** depiction of vignette dolls are found in **Fig 1, Panel B.** Holding norms in favour of TS’ was defined as a participant reporting positive attitude(s) towards others in their reference group regarding TS activity. Figurines were used to guide participant interviews and act out a vignette.

#### Qualitative analyses

The qualitative analysis methodology has been described previously [29]. Briefly, author AG digitally recorded the vignette interviews, a research assistant transcribed the interviews into Spanish, and author AG and performed translations of the transcripts from Spanish to English. AG typed observation field notes into Spanish then translated into English. Pseudonyms were given to all research participants at transcription to protect participant identity. As previously described [29], deductive thematic analysis was used: codes were organised into categories based on descriptive norms of transactional sex but other themes were allowed to emerge if data did not fit into initial themes. NVivo 12 software was used to organise and code the field notes of participant observation and individual interviews. Initial codes were generated with a deductive process: AG first read the transcripts and participant observation field notes. An initial codebook was made from our conceptual framework but was open to inclusion of new codes if they emerged, data were divided into initial codes, codes were organised into themes and a thematic map was made from the overall theme categories. Ten per cent of the transcripts were checked for inter-rater reliability with co-author MJ, translation and understanding between themes. Contradictions were agreed on with a research assistant. For this analysis, code saturation, where no new codes emerged during eight interviews in a row, was reached within the first eight coded interviews for this analysis. However, a stoppage criterion at the concomitant analysis stage was not employed and the process was completed for all interviews [31]. Interview transcriptions and fieldnotes from participant observation were triangulated to interpret the qualitative research findings.

#### Quantitative methods

The quantitative research methods have already been described elsewhere [32]. In brief, we used a two-stage cluster sampling design, where the 20 largest public high-schools (comprising 41.7% of all CNB high-school students) were ordered in decreasing size, and a systematic selection process (1 in 2) was used until ten schools were selected; the same process was used at the classroom level until we estimated 700 participants could be included; assuming two-thirds would accept approximately 900 students and guardians were approached [32]. Eligible participants were all 14–19 years, attending the chosen classes within the selected schools. Consenting or assenting (following parent/guardian consent) adolescents were asked to complete a self-administered questionnaire designed with Kobo Toolbox Software (Harvard Humanitarian Initiative, MA, USA), which was loaded onto tablets. Participants were also asked to provide blood and urine samples to test respectively for serological STIs (HIV/syphilis, all participants), and genital STIs (*Chlamydia trachomatis* and *Neisseria gonorrhoeae* [CT/NG] using molecular methods) among those who reported past sexual activity or had tested positive for a serological STI [32].

The questionnaire was adapted from previously used questionnaires written in Spanish in similar surveys in Panama [33,34]. The self-administered questionnaire was piloted for acceptability, understanding, and ease of tablet use among participants in a school that was not selected in our sampling [32]. The following variables were included: sociodemographic data (gender, age, district of study/residence), household-level economic indicators (i.e., roof material [grass/palm fronds/fabric/tarp], presence of latrine/flush toilet), and sexual behaviour history (penetrative sexual experience, number of lifetime sexual partners, sexual activity in previous 4 weeks, reported condom use at last sexual intercourse, lifetime experience of same-sex sex, and lifetime experience of forced sex). STI infections were categorised as serological (HIV/syphilis) and mucosal (chlamydia/gonorrhoea), corresponding respectively to cumulative and recent exposure status. The outcome variables related to TS were defined as *‘Ever been offered goods (money/food/housing/transport/clothing/grades) in exchange for sex’*, with follow-on questions clarifying what had been offered, who offered it and whether the offer had been accepted. Social norms questions included *‘What would your friends think if you were in the situation of being offered TS*?*´*; ´*Would you accept a better grade or money from your (man/woman) teacher or from an older (adult) man/woman in exchange for having sex with him/her*?*´;* ´*Do you think your friends would think positively or accept [that the situation is right for you] or negatively or not accept [that the situation is bad for you]*?´, ´*Would they try to stop you from doing it*?*’*.

#### Quantitative analyses

Filled questionnaires were uploaded into the Kobo Toolbox cloud, imported, and analysed in Stata V.15.0 (StataCorp, Tx, USA). Some participants did not respond to all questions, and these missing responses were not included in the analyses. Consequently, denominator values may differ by variable. The χ^2^ test with p-values was used to test statistical differences by gender in responses to normative expectations in favour or against TS. Furthermore, risk factor analyses were undertaken for four TS-associated outcomes: i) TS offer; ii) TS acceptance; iii) norms in favour of TS with an older man; and iv) norms in favour of TS with an older woman (for male participants only), using two approaches: an unadjusted analysis of demographic variables only, and an age and sex-adjusted analysis of sexual behaviours/presence of biological STI (HIV/syphilis and CT/NG analysed separately as mentioned).

We used random-effects logistical regression analysis to account for school-level clustering to analyse factors associated with TS, odds ratios (OR) and 95% confidence intervals (CI) for associations between each of the TS-associated outcomes. Variables associated with TS outcomes at p<0.2 in the univariable analysis were included in initial multivariable models, adjusting for age and gender. In the initial multivariable model, distal (sociodemographic) variables were included first, and then proximal variables (sexual behaviour and STIs) were included. The final model included all variables associated with TS-outcomes at p<0.1. Final statistical significance in the multivariable model was set at p<0.05.

### Ethical approval

The research was approved by the *Comité Nacional de Bioética de la Investigación de Panamá* (EC-CNBI-2016-05-25, November 2017), and the London School of Hygiene & Tropical Medicine (Ref:14558; January 2018). All minor participants (14–17 years) had written guardian consent and written individual assent. Participants aged 18/19 signed their own consent form. All ethical concerns were brought to the attention of the pertinent authorities.

## Results

### Qualitative findings

We identified three overall themes relating to transactional sex (TS): 1) who engages in TS, 2) negotiations and decision-making power to accept or decline a TS offer, and 3) Social rewards and sanctions.

#### Who engages in transactional sex?

Most adolescents reported that they would accept a transaction for sex with older men and women if offered. Nearly all participants mentioned that it was common for boy and girl adolescents to engage in sex with older men in exchange for money, transport, a good, or a better grade. Unchi notably said: “Of course Mario would go with the man… the majority of boys these days have sex for things…They can receive 60 dollars, or food, shoes, a cell phone…it happens a lot around here.” Bechi said, “Girls sometimes start [having sex] before they develop (menarche). Here teachers and older men [come] to the community, and they give girls what they need…girls don’t say no". Both participants agreed that older men from outside their community commonly offered TS.

Although not mentioned as frequently, some participants said that older women and female teachers in the community offer things to adolescents in exchange for sex. Unchi and Joti indicated that boys in their class had had sex to get money from older women working in their community, “especially if the boy needs the money”. Female teachers also offered TS. For example, Bechi said: “I have a friend (girl) who was not doing well in Maths class… her (woman) teacher invited her over to her house to ’practice mathematics’, the teacher said if she went, she would pass her in the class… when she got there, (my friend) realised the teacher wanted to teach her other things [*laughter*]…”. Bechi then indicated that her friend left the situation without engaging in sex. However, her (math) grade suffered from not accepting the offer.

#### Negotiations and decision-making power to engage in transactional sex

Like Bechi (above), other adolescents also felt they could refuse to engage in TS. Reasons for declining TS included 1) loving their boy/girlfriend, 2) not being interested in the person offering, 3) believing that the person offering had an STI, and 4) wanting more than what was offered. The most cited motivation for declining a TS offer was the person’s romantic ties to someone else. Joti, Olí, Jochi, Chotikö, Melikän, Bechi, Gebi and Merisi all mentioned that if *Mario* and *Juanita* cared for their boy/girlfriend, they would not engage in TS. A second reason that adolescents mentioned why girls would not have sex with an older man is if they did not like the man romantically. Mechi, Bechi and Belikan said that girls in their community would only have sex with the professor if they liked him (romantically). Otherwise, “she would say no” -Mechi, Bechi and Belikan. The third reason for declining TS was due to the presumed poor sexual health or an STI of the person offering. For example, Unchi and Olí said that the boy would not go with the older man if he offered because the man may have “something wrong down there”–Unchi and Olí. The fourth reason to decline is when what is offered is felt to be insufficient for the exchange. Chötiko, Merisi, and Chirä said that adolescents could decide about what is being offered, and if it is not enough or not what they want, they would refuse.

Although some adolescents indicated that they felt they had agency in the decision to engage in the TS offer, other times, adolescents felt that declining TS was difficult due to economic circumstances. For example, Comenchi, Melikän, Mego, Unchi, and Tächi all indicated that they would accept if the adolescents were in great need of money or gifted item. Therefore, the need for what is offered would increase the pressure to accept the offer. Tächi added, "*Mario* isn’t gay, but if he needs the food for his family, then he’ll do it.”

Other participants from each community, including Merisi, Joti and Tachi, indicated they felt that the sum of adolescent behaviour could collectively encourage or discourage TS. For example, Merisi said that if one accepted the transaction, the teachers would then believe that other adolescents also wanted to participate in TS: “The more we say ‘Yes’ to them, the more they are going to offer”. Joti and Tachi also mentioned the collective power of adolescents. Joti and Tachi similarly said that because many adolescents say yes, then adults keep offering. For example, Joti said, “if adolescents say no, then they [the adults] won´t think to ask us for sex anymore.”

#### Social rewards and punishment for engaging in transactional sex

Social sanctions, the beliefs that reward or punish specific behaviours, were investigated. Sanctions were measured by asking about friends’ opinions: *Maria* and *Juan* (*Juanita’s* or *Mario’s* friends, respectively) (Fig 1).

For boys and girls, three types of sanctions were found: 1) positive/supportive; 2) accommodating (negative sanctions that, after describing the reason for doing it, turned to acceptance of the act); and 3) negative/unsupportive. Positive sanctions were social rewards for engaging in TS. For example, Mechi said: “If *Juanita*’s friends found out about her being with the teacher, then they wouldn’t be upset that she did it; they would want to be like her and get help with school too”. Buche, Merisi, Chitigön and Unchi similarly thought *Juan* and *María* would think it was good that *Mario* or *Juanita* was having sex at all, no matter how it happened. Regarding accommodating sanctions, Buche, Tikän, Chitigön and Olí indicated that *Mario’s* and *Juanita*’s friends would not be supportive of their TS activity at first, but after the characters explained why there was a need, the friends became supportive of their decision.

Other participants mentioned negative sanctions/social punishment. Chötiko indicated the friends would think there is an unfair advantage: “If *Juanita*’s friends found out she didn’t pass by studying, they’d think she should be held back.” Mechi said that the age difference was important: "If I went with the older man, my friends would think that he’s too old for me." Of note is that Chötiko’s reacted to the unfair advantage and Mechi reacted to the partners age, but neither reacted negatively to the transaction.

### Quantitative survey findings

A total of 700 adolescents aged 14-19y (310 girls [45.1%], 378 boys [54.9%]) were enrolled in the study **(Table 1).** Boys were slightly older (median: 18 years, interquartile range [IQR]: 16–18) than girls (median: 17 years, IQR: 15–18).

**Table 1:** Participant characteristics and associations with having been offered transactional sex (Panel A) and having accepted transactional sex (Panel B) among school-going adolescents of the Comarca Ngäbe-Buglé, Panama, 2018.

	Total		Panel A. Have you been offered transactional sex?				Panel B. What decision was made concerning the TS offer?					
			**Yes**				**Went with the person**		**Did not go**			
	**n/N**	**%**	**n/N**	**%**	**OR** **(95% CI)**	**p*-*value**	**n/N**	**%**	**n/N**	**%**	**OR** **(95%CI)**	**p-value**
**Gender**						0.26						0.15
Boy	384/700	54.9	58/374	15.5	1		35/45	77.8	8/45	17.8	ref	
Girl	316/700	45.1	58/309	18.8	1.26 (0.84–1.88)		35/43	81.4	4/43	9.3	0.48 (0.18–1.30)	
**Age groups, years**						**0.01**						**0.04**
14–15	157/700	22.4	18/154	11.7	1		8/9	88.9	1/9	11.1	1	
16–17	222/700	31.7	32/218	14.7	1.30 (0.70–2.41)		21/22	95.5	1/22	4.6	8.67 (0.48–155.31)	
18–19	321/700	45.9	66/311	21.2	**2.04** **(1.16–3.60)**		41/51	80.4	10/51	19.6	**15.6** **(1.15–219.99)**	
**Economic indicators**						0.54						0.30
No latrine or tin roof	416/688	48.6	61/330	18.5	1		36/44	81.8	8/44	18.2	1	
Latrine or flush toilet and tin roof	360/700	51.4	55/353	15.6	1.87 (0.25–13.92)		34/38	89.5	4/38	10.5	0.49 (0.13–1.89)	
**Comarcal District**						0.34						**0.04**
Nidrini (Chiriquí)	362/700	51.7	65/351	18.5	1		38/45	84.4	7/45	15.6	1	
Kädridri (Veraguas)	128/700	18.3	16/125	12.8	0.65 (0.36–1.17)		8/8	100.0	0/11	0.0	**0.06** **(0.01–0.64)**	
Ño Kribo (Bocas del Toro)	210/700	30.0	35/207	16.9	0.90 (0.57–1.41)		24/29	82.8	5/29	17.2	1.31 (0.22–7.59)	

Notes: Denominators differ due to missing responses for some variables; OR = odds ratio; CI = confidence intervals; bolded OR denote statistical significance at p<0.05 level.

#### Characteristics of participants who have been offered transactional sex

The proportion of female and male participants who had ever been offered ‘Something in exchange for sex’ (TS) was similar (18.8% of 309 girls and 15.5% of 374 boys, p = 0.26) **(Table 1).** Of those, the majority (71.4% [60/84]) reported they had been offered money. Other goods offered included housing (8.3%), clothing (4.8%), a better grade from a teacher (4.8%), or a cell phone (1.2%). Among those who had been offered TS, 81.4% (35/43) of girls and 77.8% (35/45) of boys accepted (p = 0.38). There was no association of TS offer or TS acceptance with household-level economic indicators in unadjusted analyses (**Table 1**).

#### Norms governing transactional sex

Over a fifth of respondents (22.5% [71/316] girls and 22.9% [88/384] boys) held injunctive norms in favour (anticipation of people´s positive reaction of behaviour) of another adolescent having sex with an older man or male teacher in exchange for money/better grade, with no difference by respondent’s gender (p = 0.90) **(Table 1)**. Compared to norms favouring TS with an older man, a similar proportion of boys (22.6% [87/384]) indicated favourable norms of TS with an older woman. We found no association between holding norms in favour of TS and reporting having engaged in TS **(Table 2).**

**Table 2:** Norms in favour of having sex with an older man (Panel A) and with an older woman (Panel B) among school-going adolescents of the Comarca Ngäbe-Buglé, Panama, 2018.

	Panel A: Norms in favour of TS with an older man				Panel B: Norms in favour of TS with older woman (only male participants were asked)			
	**In favour of norm**		**OR** **(95% CI)**	**p-value**	**In favour of norm**		**OR** **(95% CI)**	**p-value**
	**n/N**	**%**			**n/N**	**%**		
**Gender**				0.39				
Girls	71/316	22.5	1				-	-
Boy	88/384	22.9	1.02 (0.70–1.49)		87/384	22.7	-	-
**Age group, years**				0.41				0.52
14–15	29/132	22.0	1		87/384	22.7	1	
16–17	42/132	31.8	1.14 (0.69–1.88)		15/69	21.7	1.37 (0.67–2.78)	
18–19	61/132	46.2	1.12 (0.70–1.79)		30/109	27.5	0.92 (0.47–1.79)	
**Economic indicators**				0.37				0.92
No latrine or tin roof	90/416	21.6	1		50/221	22.6	1	
Latrine or flush toilet and tin roof	67/272	24.6	1.18 (0.82–1.71)		36/156	23.1	1.03 (0.63–1.67)	
**Comarcal District**								0.77
Nidrini (Chiriquí)	82/362	22.7	1	0.26	45/204	22.1	1	
Kädridri (Veraguas)	23/128	18.0	0.75 (0.44–1.26)		13/56	23.2	1.07 (0.53–2.16)	
Ño Kribo (Bocas del Toro)	54/210	25.7	1.19 (0.77–1.83)		29/124	23.4	1.08 (0.63–1.84)	

Notes: Denominators differ due to missing responses for some variables; OR = odds ratio; CI = confidence intervals; bolded OR denote statistical significance at p<0.05 level.

#### Forced sex, sexual behaviour and HIV/STI status

*Norms*. In unadjusted analyses, sexual behaviours were not found to be associated with injunctive norms in favour of TS with an older man or woman (**Tables 2 and 3**). However, the age- and sex-adjusted analysis found an association between HIV/syphilis seropositivity and holding norms in favour of TS (adjusted odds ratio [AOR] 2.87, 95%CI: 1.23–6.67).

**Table 3:** Norms in favour of having sex a with an older man (Panel A) and with an older woman (Panel B) and sexual behaviours among school-going adolescents of the Comarca Ngäbe-Buglé, Panama, 2018.

	Panel A: Norms in favour of transactional sex with older MAN				Panel B: Norms in favour of TS with older WOMAN (only asked in boy participant questionnaires)			
	**In favour of the norm**		**OR** **(95% CI)**	**p-value**	**In favour of the norm**		**OR** **(95%CI)**	**p-value**
	**n/N**	**%**			**n/N**	**%**	**n/N**	**%**
Sexually Experience*				0.87				0.47
No	38/164	23.2	1		21/82	24.1	1	
Yes	121/536	22.6	0.97 (0.67–1.47)		66/302	21.9	0.81 (0.46–1.43)	
**Number of lifetime sexual partners**				0.39				0.81
1	30/153	19.6	1		16/76	21.1	1	
2	16/67	23.9	1.28 (0.64–2.56)		5/39	12.8	0.55 (0.18–1.64)	
3 or more	27/113	23.9	1.29 (0.71–2.32)		18/80	22.5	1.1 (0.51–2.33)	
**Currently sexually active**				0.45				0.52
No	71/328	21.7	1		39/184	21.2	1	
Yes	73/301	24.3	1.15 (0.79–1.68)		40/166	24.1	1.18 (0.71–1.95)	
**Same-sex sex partner**				0.38				0.86
No	83/359	23.1	1		43/207	20.8	1	
Yes	6/36	16.7	0.66 (0.26–1.67)		5/26	19.2	0.91 (0.32–2.55)	
**Reported condom use at last sexual intercourse**				0.56				0.69
Never	57/247	23.1	1		29/146	19.9	1	
During part of the time	10/48	20.8	0.88 (0.41–1.87)		5/30	16.7	0.81 (0.28–2.29)	
During the whole act	3/17	17.7	0.71 (0.20–2.60)		0/3	0.0	-	-
**Has experienced forced sex**				0.61				**0.10**
No	83/371	22.4	1		44/232	23.7	1	
Yes, at least once	33/132	25.0	1.13 (0.71–1.81)		7/53	13.2	0.49 (0.21–1.15)	
**Has been offered TS**				0.34				0.87
No	128/567	22.6	ref		74/316	23.4	1	
Yes	31/116	26.7	1.24 (0.79–1.97)		13/58	22.4	0.94 (0.48–1.85)	
**What did the person do when they were offered TS**				0.34				0.30
Went with the person	21/70	30.0	1		4/13	30.8	1	
Didn’t go with the person	2/12	16.7	2.31 (0.41–13.14)		6/13	46.2	0.94 (0.48–1.85)	
**Chlamydia and/or gonorrhoea infection**				**0.17**				0.43
No	110/463	23.8	1		60/271	22.1	1	
Yes	11/67	16.4	0.62 (0.31–1.23)		4/26	15.4	0.64 (0.21–1.93)	
**HIV and/or active syphilis**				**0.01**				0.22
No	106/503	21.1	1		57/278	20.5	1	
Yes	11/25	44.0	**2.89** **(1.26–6.65)**		9/21	42.9	**2.91** **(1.17–7.24)**	

*reported ever having sex or was seropositive for HIV, syphilis, HSV-2, or HBsAg, ** adjusted for age and sex. Note: Denominators differ due to missing responses for some variables; OR = odds ratio; CI = confidence intervals; Bolded OR are significant at p<0.05 level.

*Transactional sex offer*. TS offer was associated with having had sex with a same-sex partner (36.1% versus 15.0% among those not reporting same-sex sex, AOR 2.23, 95%CI: 0.91–5.47, p = 0.08). Reported forced sex was also associated with TS offer (25.7% among those who were offered TS versus 9.9% among those who were not, AOR 5.75, 95%CI: 2.87–11.52). In a separate analysis, those who reported forced sex were not more likely to be HIV/syphilis seropositive (p = 0.21). However, TS offer was associated with HIV/syphilis seropositivity (33.3% among seropositive had been offered TS versus 14.9% among seronegative, AOR 2.81, 95%CI: 1.18–6.72) **(Table 4).**

**Table 4:** Sexual behaviour and associations with having been offered transactional sex (Panel A) and having accepted transactional sex (Panel B) among school-going adolescents of the Comarca Ngäbe-Buglé, Panama, 2018.

	Panel A: Been offered transactional sex						Panel B: What participant did when TS was offered							
	Was offered transactional* sex		**OR** **(95% CI)**	**p-value**	**AOR** **(95% CI)**	**P-value**	**Went with the person**		**Didn’t go with the person**		**OR** **(95% CI)**	**p-value**	**AOR** **(95% CI)**	**p-value**
	**n/N**	**%**					**n/N**	**%**	**n/N**	**%**				
Sexually experienced ***				**0.03**		**0.03**					-	-	-	-
No	18/160	11.3					0/8	0.0	8/8	100.0				
Yes	98/523	18.7	**1.82** **(1.06–3.11)**		**1.85 **(1.08–3.17)**		62/80	83.8	12/80	16.2				
**Total number of sex partners in lifetime**				**0.03**		0.32						0.38		
1	19/150	12.7	1				15/18	83.3	3/18	16.7	1			
2	16/67	23.9	2.16 (1.03–4.53)		*1.91 **(0.93–3.93)**		13/16	81.3	3/16	18.8	0.85 (0.11–6.10)			
3 or more	25/109	22.9	2.05 (1.06–3.96)		**2.14** **(1.13–4.03)**		16/22	72.7	6/22	27.3	2.62 (0.40–17.30)			
**Currently sexually active**				**0.04**		**0.08**						0.32		
No	26/211	12.3	1		**1**		25/27	92.6	2/27	7.4	1			
Yes	50/259	19.3	**1.70** **(1.02–2.85)**		**1.96** **(0.92–4.19)**		40/49	81.6	9/49	18.4	2.42 (0.43–13.64)			
**Same-sex sex partner**				**<0.01**		**0.08**						**0.06**		**0.07**
No	52/347	15.0	1		**1**		43/49	87.8	6/49	12.2	1		1	
Yes	13/36	36.1	**3.21** **(1.53–6.73)**		**2.23** **(0.91–5.47)**		8/13	61.5	5/13	38.5	**5.06** **(0.95–27.00)**		**4.66** **(0.90–25.90)**	
**Reported condom use at last sexual intercourse**				0.84								0.44		
Never	47/242	80.6	1				35/44	79.6	9/44	20.5	1			
During part of the time	6/47	12.8	0.61 (0.24–1.51)				4/6	66.7	2/6	33.3	-			
During the whole act	4/16	25.0	1.38 (0.43–4.48)				3/3	100.0	0/4	0.0	0.15 (0.00–6.28)			
**Has experienced forced sex**				**<0.01**		**<0.01**						**0.05**		**0.05**
No	33/363	90.9	1		1		35/37	94.6	2/37	5.4	1		1	
Yes, at least once	45/126	35.7	**5.56** **(3.33–9.26)**		**5.75** **(2.87–11.52)**		26/32	81.3	6/32	18.8	**5.03** **(1.01–25.37)**		**5.93** **(1.12–31.25)**	
**CT/NG infection**				0.82										
No	70/442	15.8	1				53/65	81.5	12/65	18.5	1			
Yes	9/61	14.8	0.92 (0.43–1.95)				8/8	100.0	0/8	0.0	0.67 (0.04–11.80)	0.79		
**HIV and/or active syphilis**				**0.02**		**0.02**								
No	71/477	14.9	1		1		54/65	83.1	11/65	16.9	1			
Yes	8/24	33.3	**2.86** **(1.18–6.93)**		**2.81** **(1.18–6.72)**		7/8	87.5	1/8	12.5	0.62 (0.06–6.41)	0.69		

*Totals per variable differ due to missing responses. ** Independent correlate *** reported to have had sex or tested positive for HIV, syphilis, HSV-2 or HBsAC

Note: Denominators differ due to missing responses for some variables; OR = odds ratio; CI = confidence intervals; Bolded OR are significant at p<0.05 level‥

*Acceptance of transactional Sex*. In age-sex adjusted analyses, those who declined the offered TS were more likely to report forced sex (18.8% versus 5.4% among those who did not report forced sex, AOR 3.11, 95%CI: 1.09–9.86) **(Table 4).**

## Discussion

In this study, we used a mixed methods design, sequentially collecting qualitative and quantitative data to describe the characteristics and norms of transactional sex among indigenous adolescents living in the Comarca Ngäbe-Buglé (CNB), in one of the poorest areas of Panama. We found that TS was a relatively common and socially well-accepted experience among CNB adolescents, similar to studies among similar populations in other low- and middle-income countries (LMIC) [4,12,35]. Participants in the qualitative study reported that older men and women often offer TS to school-going adolescents. Normative beliefs in acceptance of TS were not associated with participant sex, age, or household-level socioeconomic status. Whilst most adolescents felt they could easily accept or decline a TS offer, some had limited agency when in extreme need of what was being offered. We found that norms and reported behaviours towards TS were associated with HIV/syphilis serostatus of participants and, disturbingly, the experience of forced sex. Qualitative and quantitative results will be integrated in interpretation below, by using weaving approach to write qualitative and quantitative results together in the following four overarching points of reflection: 1) general characteristics of TS, 2) economic and social factors related to TS, 3) forced sex and TS, and 4) HIV/STI outcomes related to TS.

### General characteristics of transactional sex

Nearly one in six adolescents in CNB (almost 20% of girls and 15% of boys) reported having been offered TS; the vast majority who were offered TS, accepted the offer (81% of girls, 72% of boys). These rates are much higher than reported by adolescents in urban areas of Panama (4.8% by girls and 4.3% by boys) [36], and comparable to reports by adolescent girls in sub-Saharan Africa [7,9], underscoring the extreme vulnerability of adolescent boys and girls. Surprisingly, the experience of TS was similar in girls and boys, whilst elsewhere there is a gendered experience of TS by income setting with a predominance showing girls in LMICs, and boys in high-income countries report TS behaviours, as reported by a systematic review [35].

#### Economic and social factors related to transactional sex

No household-level economic variables were associated with TS acceptance in the quantitative analysis. However, our qualitative study revealed that an individual’s economic need did influence the decision to engage in sex. This discrepancy may have occurred because of the asymmetrical focus on household-level poverty in the quantitative study and individual needs in the qualitative study. The CNB has the highest poverty levels in Panama, and the distribution of poverty within is relatively homogenous [24], thereby erasing the ability of identifying particular household indicators, collected in our quantitative questionnaire. Some studies have indicated that in circumstances of extreme poverty, survival sex or sex to meet basic economic needs is practised by young people [24,37–39], although this is not found in all studies [35,40,41]. Future research would need to focus on individual and household economic factors that drive TS activity among CNB adolescents.

Social rewards and punishments regarding TS appeared to embrace the entire spectrum, from being positive/supportive, accommodating, or negative/unsupportive. Despite the importance of measuring social sanctions that may be holding a TS behaviour in place, sanctions have not been extensively studied in TS research worldwide. Two exceptions are studies in Tanzania, which found negative sanctions on young women who do not receive something in exchange for sex, and sexual abuse may be perceived if a gift was not given [42,43]. In contrast to these studies, we did not elicit material expressions of love in TS; the social rewards participants sought were related to school achievement and fairness.

Our qualitative survey findings suggest that CNB adolescents feel a sense of agency in their decision to engage in TS. Participants indicated that individually adolescents could negotiate to receive something they wanted or to accept or decline based on what is being offered. Additionally, collectively adolescents have power in the market supply of TS; if adolescents decline TS offers, those offering will be less inclined to offer to others. Some scholars believe TS itself is associated with acting with agency, where the person receiving the goods in exchange for sex are working to access power and resources [44,45]. A study in Swaziland demonstrated that empowerment could lead to better-negotiated condom use or leaving a TS partner when the situation felt unsafe [2].

#### Forced sex and transactional sex

Participants who had been offered TS, and worryingly those who declined TS offers were more likely to have reported forced sex. Due to the cross-sectional nature of the quantitative study, the temporal association could not be determined; it is unknown whether declining TS led to forced sex or whether those who experienced forced sex were more likely to decline TS afterwards. A history of childhood sexual abuse has been correlated with a history of exchange of sex [9,35,46,47]. The study in Swaziland found that the link between partner violence and TS was strongly correlated with the adolescents’ limited agency in the decision to have sex [48]. The limited agency was found to be more influenced by broader dynamics of poverty, hunger, fear, violence, and hegemonic masculinities, than the transaction of money or gift giving [48]. A Latin American multi-country study among MSM populations found that TS encounters were associated with partner violence, including forced sexual experience and verbal or physical abuse [49].

#### Transactional sex and HIV/STI outcomes

Worldwide, TS has been shown to be associated with an increased risk of HIV and other STIs [6–12]. Our study found an association between having been offered TS and seropositivity for HIV/syphilis. We also found that those who had been offered and accepted TS were more likely to report same-sex sexual activity than other adolescents. In the CNB, HIV and syphilis are concentrated in young male populations [32], which could explain some of these associations. By contrast, we did not find that chlamydia or gonorrhoea infections were associated with TS. These findings are perhaps not surprising because these infections tend to reflect more recent exposures and are more likely to be acquired in sexual encounters with other young people who have higher prevalence than adults.

#### Implications for policy and practice

Because of the tangled web of effects on TS, limited agency due to poverty and social norms, and felt agency that individuals held, programmatic interventions that focus on eliminating the transactional component of adolescent sex would not necessarily have a meaningful effect on lowering forced sex and adverse HIV/STI outcomes [48]. Instead, interventions should focus on economic and structural factors such as increasing economic empowerment and extreme poverty through improved cash transfer programmes, scholarship incentive programmes or increased employment opportunities for youth. Secondly, to tackle HIV and syphilis prevalence, there is a need to reinvigorate condom promotion campaigns that focus on increasing access to condoms and at the same time empower young people to use them in all sexual encounters, particularly during TS. These components could be included in school-based, community based, and online sexuality education programs [30]. Thirdly, considering the association between declining a TS offer and the experience of forced sex, the CNB security and judicial systems should be better supported so perpetrators are held accountable for all forms of violence. Furthermore, educational campaigns could include identification and reporting of violence, especially sexual violence for children and adolescents. Therefore, a comprehensive intervention could include sexuality education in schools and people-centred, community-guided projects, with clear, confidential, and protective support services for survivors of sexual violence and their families, as well greater legal accountability of violence perpetrators.

#### Limitations

Our study had several limitations. First, in the quantitative survey, we may have encountered participant selection bias due to the targeting of schools as a sampling frame to reach adolescents and the role of parents and guardians as gatekeepers. Secondly, reporting bias may have arisen with a self-administered questionnaire. Thirdly, a cross-sectional study design cannot directly assess the temporality of events in the adolescents’ lives, obscuring the chronological links between the occurrence of TS, forced sex and sexual health outcomes. Fourthly, the qualitative survey only included 20 participants from two sites. However, those sites were chosen from two different regions of CNB (to ensure representativeness) and were of similar size and population density as those included in the quantitative survey; they yielded consistent results between them, rapidly reaching data saturation on topics such as TS so that this limitation may have been minimised. Lastly, as this analysis was part of a larger study focusing on the epidemiology of STIs, specific variables of interest, for example measuring power dynamics (patriarchy, gender relationships, but also issues of capitalism and international power relations), comprehensive economic variables and a detailed description of how culture may influence normative TS beliefs, could not be easily collected or undertaken. Follow-up research on TS in this region should include these variables.

## Conclusions

Reported TS is a common and relatively normalised experience with low sanctions among boy and girl adolescents in CNB. People who offer TS are adults (men and women), from the community or from outside, also disturbingly including schoolteachers. Adolescents feel they generally have both individual agency and collective power in deciding about TS, although frequent adverse sexual health outcomes, including HIV/syphilis and forced sex, occur. Therefore, interventions designed to mitigate the impact of TS should focus on economic stability, empowering adolescents for informed decision-making, increasing awareness and knowledge of forced sex and sexual health risks, and increasing the access and use of condoms in all sexual encounters. In addition, safe reporting mechanisms should be developed, and perpetrators must be held legally accountable for sexual violence.

## Supporting information

S1 Checklist STROBE statement—checklist of items that should be included in reports of observational studies. (DOCX)
