# Supplementary material for: Social norms that sustain transactional sex and associations with sexual health outcomes: A mixed-methods study in the Comarca Ngäbe-Buglé, a rural-Indigenous region of Panama
Source: PLoS One. 2024 May 31;19(5):e0304805. doi: 10.1371/journal.pone.0304805 (PMC11142491; doi:10.1371/journal.pone.0304805)
Supplement: S1 Checklist — (DOCX) [file pone.0304805.s001.docx]

STROBE Statement—checklist of items that should be included in reports of observational studies

|  | Item No. | Recommendation | Page  No. | Relevant text from manuscript |
| --- | --- | --- | --- | --- |
| **Title and abstract** | 1 | (*a*) Indicate the study’s design with a commonly used term in the title or the abstract | 2 | `We conducted a mixed-methods study in the CNB between January and November 2018, which included (i) semi-structured interviews with adolescents focusing on descriptive norms related to TS; (ii) a two-stage cluster sample design was used to select public-school-going adolescents` |
|  |  | (*b*) Provide in the abstract an informative and balanced summary of what was done and what was found | 2 | Whole abstract |
| Introduction | | | |  |
| Background/rationale | 2 | Explain the scientific background and rationale for the investigation being reported | 3 | ´ page 3-4´ |
| Objectives | 3 | State specific objectives, including any prespecified hypotheses | 3 | ´ to describe transactional sex among adolescents, the normative beliefs surrounding TS activity and the association of TS with adverse sexual health outcomes´ |
| Methods | | | |  |
| Study design | 4 | Present key elements of study design early in the paper | 4 | ´In this article, we integrated qualitative and quantitative research in two ways: a) at the design level and b) at the interpretation level. At the design level, as little was known about the sexual behaviours and STIs of adolescents of the CNB, an exploratory sequential framework was used where qualitative methods were used first; quantitative methods were then used to measure the generalisability and prevalence of qualitative findings and STI outcomes |
| Setting | 5 | Describe the setting, locations, and relevant dates, including periods of recruitment, exposure, follow-up, and data collection | 4-7 | See pages 4-7 |
| Participants | 6 | (*a*) *Cohort study*—Give the eligibility criteria, and the sources and methods of selection of participants. Describe methods of follow-up  *Case-control study*—Give the eligibility criteria, and the sources and methods of case ascertainment and control selection. Give the rationale for the choice of cases and controls  *Cross-sectional study*—Give the eligibility criteria, and the sources and methods of selection of participants | 5 | ´ Ethnographic observation was used during the first ten days in each community to purposively select twenty 14-19-year-old public-school-going adolescents (five girls and five boys from each community) for interviews using vignettes to elicit descriptive norms and social sanctions….. Eligible participants were all 14-19 years, attending the randomly chosen classes within the selected schools.´ |
|  |  | (*b*) *Cohort study*—For matched studies, give matching criteria and number of exposed and unexposed  *Case-control study*—For matched studies, give matching criteria and the number of controls per case |  |  |
| Variables | 7 | Clearly define all outcomes, exposures, predictors, potential confounders, and effect modifiers. Give diagnostic criteria, if applicable | 7 | ´ Analyses were undertaken for four TS-associated outcomes, i) TS offer; ii) TS acceptance; iii) norms in favour of TS with an older man; and iv) norms in favour of TS with an older woman (for male participants only), using two approaches: an unadjusted analysis of demographic variables only, and an age and sex-adjusted analysis of sexual behaviours/presence of biological STI.´ |
| Data sources/ measurement | 8* | For each variable of interest, give sources of data and details of methods of assessment (measurement). Describe comparability of assessment methods if there is more than one group | 6 | Quantitative methods section |
| Bias | 9 | Describe any efforts to address potential sources of bias | 6 | Quantitative methods section |
| Study size | 10 | Explain how the study size was arrived at | 6 | Quantitative methods section |

Continued on next page

| Quantitative variables | 11 | Explain how quantitative variables were handled in the analyses. If applicable, describe which groupings were chosen and why | 7 | Quantitative analysis section |
| --- | --- | --- | --- | --- |
| Statistical methods | 12 | (*a*) Describe all statistical methods, including those used to control for confounding | 7 | Quantitative analysis section |
|  |  | (*b*) Describe any methods used to examine subgroups and interactions | 7 | Quantitative analysis section |
|  |  | (*c*) Explain how missing data were addressed | - | - |
|  |  | (*d*) *Cohort study*—If applicable, explain how loss to follow-up was addressed  *Case-control study*—If applicable, explain how matching of cases and controls was addressed  *Cross-sectional study*—If applicable, describe analytical methods taking account of sampling strategy | 7 | Quantitative analysis section |
|  |  | (*e*) Describe any sensitivity analyses | - | - |
| Results | | | | |
| Participants | 13* | (a) Report numbers of individuals at each stage of study—eg numbers potentially eligible, examined for eligibility, confirmed eligible, included in the study, completing follow-up, and analysed | 8 and 12 | First lines of qualitative and quantitative result sections |
|  |  | (b) Give reasons for non-participation at each stage | - | - |
|  |  | (c) Consider use of a flow diagram | - | - |
| Descriptive data | 14* | (a) Give characteristics of study participants (eg demographic, clinical, social) and information on exposures and potential confounders | 8-14 | Qualitative and quantitative results |
|  |  | (b) Indicate number of participants with missing data for each variable of interest | - | - |
|  |  | (c) *Cohort study*—Summarise follow-up time (eg, average and total amount) |  |  |
| Outcome data | 15* | *Cohort study*—Report numbers of outcome events or summary measures over time |  |  |
|  |  | *Case-control study—*Report numbers in each exposure category, or summary measures of exposure | *8-14* | Qualitative and quantitative results |
|  |  | *Cross-sectional study—*Report numbers of outcome events or summary measures |  |  |
| Main results | 16 | (*a*) Give unadjusted estimates and, if applicable, confounder-adjusted estimates and their precision (eg, 95% confidence interval). Make clear which confounders were adjusted for and why they were included | Table 1 | See table 1 |
|  |  | (*b*) Report category boundaries when continuous variables were categorized | Table 1 | Table 1 |
|  |  | (*c*) If relevant, consider translating estimates of relative risk into absolute risk for a meaningful time period | - | - |

Continued on next page

| Other analyses | 17 | Report other analyses done—eg analyses of subgroups and interactions, and sensitivity analyses | Table 4-5 | Table 4 and 5 |
| --- | --- | --- | --- | --- |
| Discussion | | | | |
| Key results | 18 | Summarise key results with reference to study objectives | 19 | First paragraph of discussion |
| Limitations | 19 | Discuss limitations of the study, taking into account sources of potential bias or imprecision. Discuss both direction and magnitude of any potential bias | 23 | Limitations section |
| Interpretation | 20 | Give a cautious overall interpretation of results considering objectives, limitations, multiplicity of analyses, results from similar studies, and other relevant evidence | 23-24 | Discussion section |
| Generalisability | 21 | Discuss the generalisability (external validity) of the study results | 23-24 | Discussion section |
| Other information | |  | | |
| Funding | 22 | Give the source of funding and the role of the funders for the present study and, if applicable, for the original study on which the present article is based | 28 | Funding details section |

*Give information separately for cases and controls in case-control studies and, if applicable, for exposed and unexposed groups in cohort and cross-sectional studies.

**Note:** An Explanation and Elaboration article discusses each checklist item and gives methodological background and published examples of transparent reporting. The STROBE checklist is best used in conjunction with this article (freely available on the Web sites of PLoS Medicine at http://www.plosmedicine.org/, Annals of Internal Medicine at http://www.annals.org/, and Epidemiology at http://www.epidem.com/). Information on the STROBE Initiative is available at www.strobe-statement.org.
